# Identifying Key Factors in Predicting Chikungunya and Zika Transmission Across French Polynesia and the French West Indies: A Data-Driven Probabilistic and Mathematical Modeling Approach

**DOI:** 10.1007/s11538-026-01728-x

**Published:** 2026-08-19

**Authors:** Zhiyuan Yu, Xi Huo, Peter J. Thomas, Qimin Huang

**Affiliations:** 1 Department of Bioinformatics, University of Michigan, Ann Arbor, MI 48109 USA; 2 Department of Mathematics, University of Miami, Coral Gables, FL 33146 USA; 3 Department of Mathematics, Applied Mathematics, and Statistics, Case Western Reserve University, Cleveland, OH 44106 USA; 4 Department of Data and Computer Science, Case Western Reserve University, Cleveland, OH 44106 USA; 5 Department of Mathematical and Computational Sciences, The College of Wooster, Wooster, OH 44691 USA

**Keywords:** Data-driven mathematical model, Chikungunya, Zika, Parameter estimation, Disease forecasting, Vector control

## Abstract

Chikungunya (CHIKV) and Zika are arboviruses transmitted by ectothermic *Aedes* mosquitoes, leading to seasonal outbreak patterns of viral infections. Mathematical models linked with vector and case data have proven powerful for understanding arboviral transmission dynamics, but it remains unclear if and which environmental, vector, and host features are essential for accurately capturing transmission dynamics and short-term forecasting. We began with a baseline modified SEIR compartmental model, extended it into a weekly probabilistic model aligned with reported surveillance data, and evaluated variants that include or exclude mosquito dynamics (M), temperature effects (T), and precipitation effects (P). We calibrated these variants to weekly CHIKV and Zika incidence, temperature, and precipitation data from nine regions in French Polynesia and the French West Indies. We used AIC-based model comparison to assess goodness-of-fit, rolling-window forecasting to compare predictive performance with statistical and null baselines, and the best-fitting model to estimate biologically meaningful transmission parameters. Incorporating mosquito dynamics, temperature, and precipitation substantially improves prediction accuracy, with precipitation acting as the primary environmental driver and temperature serving as a modulator. The corresponding SEI+M+T+P formulation achieves forecasting accuracy comparable to ARIMA while providing mechanistically interpretable predictions. CHIKV possesses higher transmissibility than Zika, with regionally heterogeneous parameters driven by varying mosquito-human transmission rates and mosquito densities, whereas Zika parameters are relatively homogeneous across regions. These findings suggest uniform vector control may be effective for Zika, whereas region-specific strategies are needed for CHIKV. Sensitivity analysis indicates that rapid diagnosis and isolation of symptomatic individuals are among the most effective measures for reducing transmission of both viruses.

## Introduction

The term arbovirus refers to a type of virus spread to people by the bite of blood-feeding insects such as mosquitoes and ticks (Gubler 2001). Chikungunya (CHIKV) fever and Zika fever are diseases caused by arboviral infections, and such viruses are transmitted via mosquitoes of the *Aedes* genus (Richard et al. 2016). In recent years, these two diseases have spread quickly across the world, and several large outbreaks have occurred in the regions of India, the Pacific Ocean, and South America (Riou et al. 2017). In addition, arboviruses are likely to expand to other countries via the travel of asymptomatic individuals. Predictive and inferential models of arboviral disease outbreaks can assist public health authorities in raising awareness in local communities, making travel recommendations, and conducting effective vector control interventions. These preventative measures could help slow the rate of transmission and minimize the number of outbreaks.

Mathematical modeling of vector-borne transmission dates back to the foundational compartmental and vector-host frameworks developed for malaria, including the work of Ross, Kermack–McKendrick, and the Ross–Macdonald formulation (Ross 1911; Kermack and McKendrick 1927, 1932, 1933; Macdonald 1952; Anderson and May 1979). Building on these classical models, recent work has focused on balancing mechanistic realism with statistical identifiability by extending SEIR-type structures to include explicit vector infection dynamics, stochasticity, and environmental forcing. Examples include stage-structured or vector-explicit models used to reconstruct CHIKV outbreaks (Yakob and Clements 2013), comparative modeling of dengue and Zika transmission (Funk et al. 2016), optimal control formulations for Zika (Bonyah and Okosun 2016), and models that incorporate weather-dependent mosquito ecology and population structure (Chen et al. 2023; Brozak et al. 2022; Ibrahim and Dénes 2023). A central inferential target in many such models is the basic reproduction number ($R_0$), which summarizes transmission potential in a nearly susceptible population (Wiratsudakul et al. 2018).

While complex models can capture more detailed biological mechanisms, they may introduce many parameters that are difficult to identify from limited surveillance data. Conversely, purely statistical time-series models can be effective for short-term prediction but often provide limited mechanistic interpretability and may omit intrinsic drivers such as vector dynamics (Imai et al. 2015; Unkel et al. 2012). Motivated by this trade-off, we develop a mechanistic framework that remains parsimonious while combining an SEIR-type transmission structure with stochasticity and both intrinsic and extrinsic drivers of transmission. Here, by mechanistic, we mean that all model compartments and parameters correspond to explicit biological processes, rather than being constructed solely to fit observed incidence patterns statistically. By parsimonious, we mean that the model retains only those mechanisms needed to capture the observed dynamics and support reliable inference, avoiding unnecessary complexity that is not identifiable from the available data. In particular, we model transmission as a stochastic process shaped by intrinsic factors (e.g., susceptible depletion and vector-mediated infection dynamics) and extrinsic covariates (e.g., temperature and precipitation), using the inferred parameters to estimate biologically interpretable quantities such as the basic reproduction number. Throughout the manuscript, statistical time-series models are used as forecasting baselines, whereas the probabilistic compartmental models are used to represent transmission mechanisms and estimate biologically interpretable parameters.

Mosquitoes acquire infection by biting infected humans and later transmit the virus to susceptible hosts, making mosquito abundance and infection dynamics essential components of compartmental models of arbovirus outbreaks. For example, most previous studies that revisit past arbovirus outbreaks have incorporated mosquito population dynamics (Hladish et al. 2018; Metelmann et al. 2021; Poletti et al. 2011; Caldwell et al. 2021; Leach et al. 2020; Li et al. 2019; Yi et al. 2019). On the other hand, some models targeting ongoing outbreaks may interpret the case data without incorporating mosquito population dynamics (Gao et al. 2016). This motivates an explicit comparison between model variants with and without mosquito dynamics, as well as a subsequent assessment of whether additional exogenous drivers (e.g., weather factors) provide predictive value beyond vector mediation.

This study investigates the roles of mosquito population dynamics and local weather covariates in arbovirus outbreaks. We consider a single SEI/SEIR-type compartmental modeling framework and extend it to a weekly probabilistic formulation to align with the weekly surveillance data. We then modify this stochastic formulation into different variants by including or excluding mosquito dynamics (M), temperature forcing (T), and precipitation forcing (P). We apply these variants to outbreak data for CHIKV and Zika fevers, together with local temperature and precipitation, across nine regions in French Polynesia and the French West Indies. We calibrate these variants and compare them to identify a parsimonious formulation that balances interpretability with goodness-of-fit and predictive performance.

From an applied standpoint, forecasting short-term incidence is essential for situational awareness and timely vector control. Accordingly, in addition to calibrating models to reconstruct past outbreaks, we evaluate their out-of-sample predictive performance via rolling-window forecasting and cross-disease validation using dengue incidence data from Brazil. In particular, we compare mechanistic forecasts generated by our compartmental models against standard statistical forecasters (ARIMA and exponential smoothing) and simple null baselines (historical mean, linear autoregression, and quadratic autoregression). This design allows us to assess whether incorporating transmission structure and biologically meaningful parameters yields improved predictive skill beyond purely autoregressive trend-following methods, while retaining interpretability.

Our modeling framework explicitly incorporates environmental covariates (temperature and precipitation) as exogenous drivers that can modulate vector-borne transmission (Reinhold et al. 2018; Mordecai et al. 2017; Huber et al. 2018). Mosquitoes are ectothermic, meaning their reproduction, development, feeding, and survival rates are sensitive to ambient temperature, and hence local temperature fluctuations can translate into time-varying transmission potential (Reinhold et al. 2018; Mordecai et al. 2017). Temperature influences mosquito development and survival, as well as biting activity and pathogen development within the vector (Reinhold et al. 2018; Mordecai et al. 2017); precipitation affects the availability of aquatic breeding sites and thus can alter mosquito abundance and, indirectly, the force of infection (Abiodun et al. 2017; Li et al. 2019). Since arbovirus outbreaks often occur in warm seasons and in subtropical and tropical settings, it is not always clear whether incorporating temperature and precipitation improves inference and forecasting beyond what is captured by mosquito dynamics alone. Motivated by these mechanisms, we consider model variants in which temperature and precipitation enter the transmission system (potentially with time lags), and we quantify whether these additions improve model fit and predictive performance relative to models without weather forcing.

Although our main empirical focus is on CHIKV and Zika in French Polynesia, the proposed approach is intended as a portable framework for *Aedes*-borne arboviruses more broadly. Dengue, CHIKV, and Zika share common vector ecology and are often co-circulating in similar climatic and geographic settings, making forecasting and inference tools transferable in structure (Patterson et al. 2016; Liu et al. 2020). At the same time, these viruses differ in clinically observed incidence, immunity structure, and other biological factors (Liu et al. 2020; Funk et al. 2016); thus, extending the framework to dengue or to other regions primarily requires re-parameterization and, when necessary, modest modifications to reflect pathogen-specific assumptions and data availability. In this sense, our goal is to provide a parsimonious, data-driven mechanistic modeling strategy that can be adapted across arboviruses and locations while supporting principled model comparison and out-of-sample validation.

**Fig. 1:**
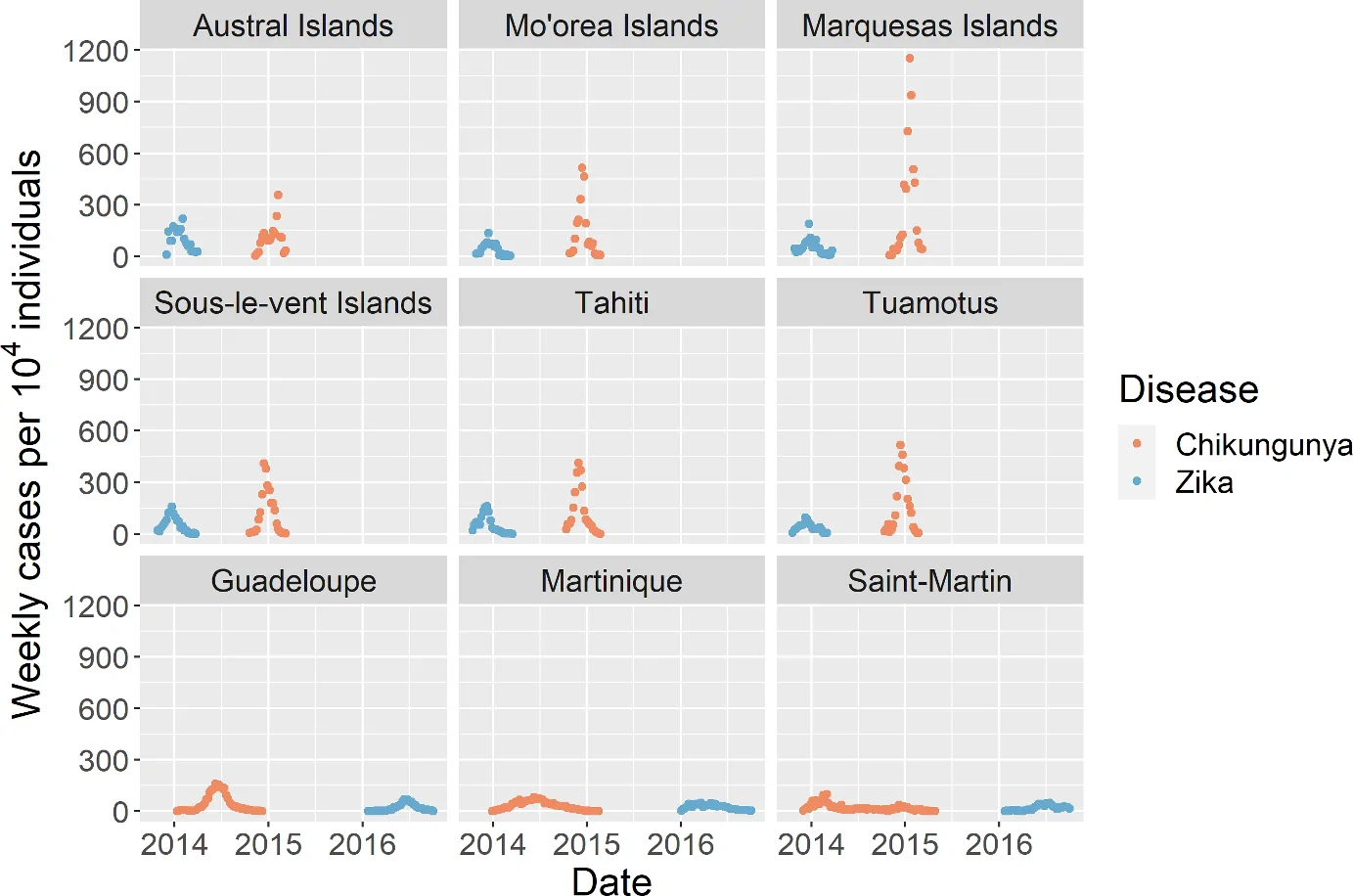
Weekly incidence normalized per 10,000 population for CHIKV and Zika across nine regions. Points indicate reported cases (CHIKV: *orange; Zika: blue*) (Color figure online)

Once we establish the optimal model, defined here as the model variant with the smallest AIC among the compared variants, we use it to address several biological questions about the outbreak data shown in Fig. 1. Firstly, although the primary mosquito species that transmit Zika and CHIKV are the same, why do most regions experience outbreaks of different magnitudes? We seek a qualitative explanation of the factors that drive different outbreak sizes of both diseases within the same region. Secondly, we investigate whether the observed similarity in Zika outbreak sizes across all nine regions indicates shared underlying transmission dynamics. Finally, we examine what factors may explain the occurrence of the largest CHIKV outbreak in the Marquesas Islands compared to other regions.

## Methods

### Data Description

The incidence data consist of successive weekly time series for CHIKV and Zika in nine regions: six islands/archipelagoes of French Polynesia (Austral, Marquesas, Mo’orea, Sous-le-vent, Tahiti, and Tuamotus) and three regions in the French West Indies (Guadeloupe, Martinique, and Saint-Martin) (Riou et al. 2017). These time series represent weekly incidence numbers reported to health practitioners, rather than the actual number of infections in the population.

Weekly average temperature (°C) is available for each region over its outbreak period (Appendix Fig. 13), and weekly total precipitation (cm) is shown in Appendix Fig. 14.

For out-of-sample validation, we additionally consider cross-disease validation using dengue incidence in Brazil retrieved from InfoDengue (InfoDengue Project 2026).

For model fitting, $Y_t$ (and $\Delta Y_t$) denotes the raw number of newly reported cases in week *t*. For visualization and between-region comparison in Fig. 1, we additionally plot incidence normalized per 10,000 population.

### Baseline ODE Model

We start with a continuous-time baseline compartment model, in which susceptible (S) and exposed/asymptomatic (E) populations follow their standard definitions. For the infectious/symptomatic (I) population, we assume that a certain fraction ($p_h$) of the newly infectious individuals would be hospitalized (H) and stop contributing to new transmissions. The H compartment is included to link the underlying transmission dynamics to the observed incidence data, which consist of reported clinical cases rather than all infections. Lastly, we assume persistent immunity after recovery (R).

1 $$\begin{aligned} \frac{dS}{dt}= &  - \beta I \frac{S}{N} \nonumber \\ \frac{dE}{dt}= &  \beta I \frac{S}{N} - \alpha E \nonumber \\ \frac{dI}{dt}= &  (1 - p_h) \alpha E - \gamma I \nonumber \\ \frac{dR}{dt}= &  \gamma (I + H) \nonumber \\ \frac{dH}{dt}= &  p_h \alpha E - \gamma H \end{aligned}$$

Because both surveillance incidence and environmental covariates are available only at weekly resolution (Fig. 1), fitting and validating the continuous-time baseline model would require further assumptions on the subweekly trajectories. Therefore, we consider the analogous discrete baseline model with a time unit of one week. The discrete equations below describe the average weekly changes in each compartment, which can be matched to weekly data (e.g. $p_h \alpha E_t$ represents the expected weekly incidence).

2 $$\begin{aligned} S_{t+1} - S_{t}= &  - \beta I_t \frac{S_t}{N} \nonumber \\ E_{t+1} - E_{t}= &  \beta I_t \frac{S_t}{N} - \alpha E_t \nonumber \\ I_{t+1} - I_{t}= &  (1 - p_h) \alpha E_t - \gamma I_t \nonumber \\ R_{t+1} - R_t= &  \gamma (I_t + H_t) \nonumber \\ H_{t+1} - H_{t}= &  p_h \alpha E_t - \gamma H_t \end{aligned}$$

Based on the continuous-time baseline ODE compartment model, its weekly discretization, and the equivalent continuous-time Markov chain formulation, we first develop the baseline stochastic weekly model. The discretization introduces a weekly tau-leaping approximation, in which transition rates are treated as approximately constant within each week. Appendix B shows that, under this approximation, the infection-count distribution has the same form as that obtained from the equivalent continuous-time Markov chain.

**Fig. 2:**
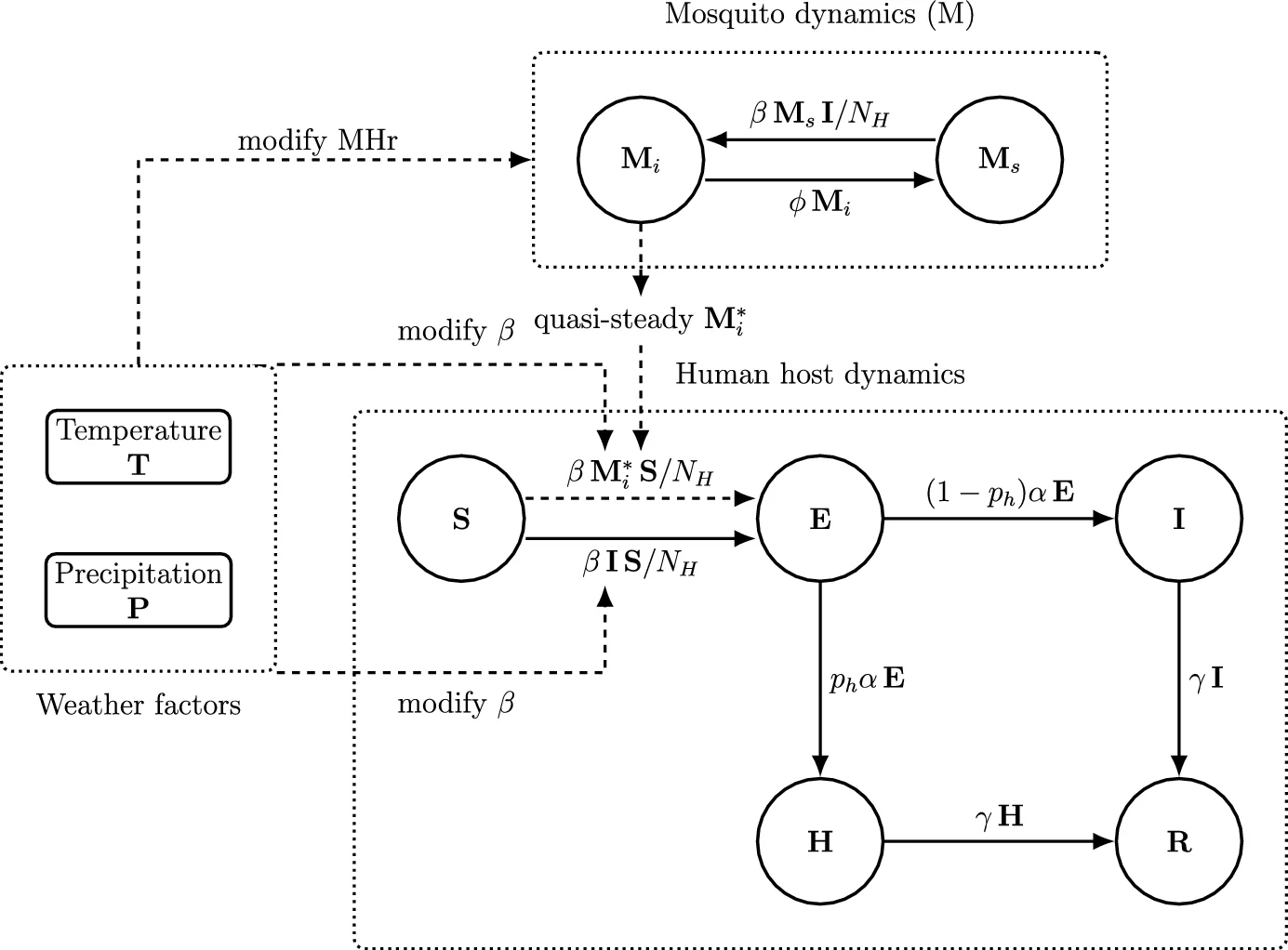
Schematic of the compartmental modeling framework. Solid arrows denote the baseline SEIRH host dynamics. Dashed arrows denote optional mosquito and weather extensions. In vector-explicit variants, mosquito dynamics contribute to the force of infection through the quasi-steady infected mosquito term $\textbf{M}_i^*$; temperature and precipitation modify the transmission rate β and mosquito-to-human ratio MHr. The eight model variants are obtained by including or excluding the mosquito component (M) and the weather effects (T and P)

We then extend this baseline with mosquito infection dynamics (M) and/or weather factors (temperature T and precipitation P) to quantify the contribution of each factor and compare model performance. A schematic of the full modeling framework is shown in Fig. 2.

### Probabilistic ODE-Derived Models

The derivation of the baseline stochastic weekly model and its variants proceeds by mapping infectious individuals to new infections and hospitalizations. The order of transitions and the rate formulas follow strictly from the baseline ODE model (1) and its weekly discretization (2), and the stochastic parts include minor modifications for the convenience of model fitting, without changing the final distributional form. Compared with the baseline ODE model (1) and its weekly discretization (2), the baseline stochastic weekly model has an explicit probabilistic form that can be fitted directly to the weekly surveillance data, and the model variants only replace some of the parameters with functions that incorporate mosquito dynamics, temperature, and/or precipitation, without changing the distributional form. This stochastic formulation captures important sources of noise between disease transmission and reported incidence, and therefore more realistically represents weekly reported case data than the deterministic ODE model while retaining the same mechanistic compartmental structure. For simplicity, we refer to the baseline stochastic model and its variants using the shorter label “SEI” from now on; the hospitalization and recovery transitions remain included in the probabilistic formulation.

#### Base Probabilistic Model (SEI)

The base model, referred to as SEI, follows the previous baseline ODE framework (1) but replaces the deterministic transitions above with stochastic counterparts to capture process noise. First, the human infectious period τ follows an exponential distribution:

$$ \tau \sim {\text {Expo}}(\gamma ). $$

As in many continuous-time compartmental epidemic models (Yakob and Clements 2013; Riley et al. 2003), we assume exponential waiting times for the exposed and infectious stages. This follows from the equivalent continuous-time Markov formulation with constant per-capita transition rates. We acknowledge that real incubation and infectious periods need not be exactly exponential and are often modeled using gamma, lognormal, or fixed-duration assumptions (Lloyd 2001; Wearing et al. 2005); however, with only weekly incidence data, such alternatives would introduce additional parameters that are not identifiable from the present data.

The number of newly exposed people (ΔE) caused by one infected individual follows a Poisson distribution, with a mean proportional to the infectious window τ and transmission rate:

$$ \Delta E \sim {\text {Poiss}}\left( \tau \beta \frac{S}{N_H}\right) , $$

where β, *S*, and $N_H$ represent the transmission rate, the size of the susceptible population, and the size of the total human population. In the equivalent continuous-time Markov formulation, infection events are treated as random transition events occurring at rate $\beta S/N_H$. Under the weekly tau-leaping approximation, this rate is treated as approximately constant within each week, so conditional on the infectious duration, the number of newly exposed individuals follows a Poisson distribution. The compound distribution is therefore geometric:

$$\begin{aligned} f_{\tau }(\tau )&= \gamma e^{-\gamma \tau } \\ f_{\Delta E|\tau }(k)&= \frac{(\tau \beta \frac{S}{N_H})^ke^{-(\tau \beta \frac{S}{N_H})}}{k!} \\ f_{\Delta E,\tau }&= \frac{(\tau \beta \frac{S}{N_H})^k}{k!} \gamma e^{-(\gamma + \beta \frac{S}{N_H})\tau } \\ f_{\Delta E}&= \int _{0}^{\infty } f_{\Delta E,\tau } \, d\tau \\&= \gamma \left( \beta \frac{S}{N_H}\right) ^k \frac{1}{(\gamma + \beta \frac{S}{N_H})^{(k+1)}} \\&= \left( \frac{\beta \frac{S}{N_H}}{(\gamma + \beta \frac{S}{N_H})}\right) ^k\frac{\gamma }{(\gamma + \beta \frac{S}{N_H})} \\ \Delta E&\sim {\text {Geom}}\left( p_r = \frac{\gamma }{\gamma +\beta \frac{S}{N_H}}\right) , \end{aligned}$$

Moreover, when there are multiple infected individuals (*I*), the total number of exposures follows a negative binomial distribution.

Let $X_1, X_2 \sim {\text {Geom}}(p_r)$ and independent of each other,

$$\begin{aligned} X&= X_1 + X_2 \\ f_X(k)&= \sum _{i = 0}^{k}f_{X_1}(i)f_{X_2}(k-i) \\&= p_r^2\sum _{i = 0}^{k}(1 - p_r)^i(1 - p_r)^{k - i} \\&= (k + 1) p_r^2(1 - p_r)^k \\&= \left( {\begin{array}{c}k + 1\\ 1\end{array}}\right) p_r(1 - p_r)^kp_r \\ X&\sim {\text {NB}}(r = 2, p = p_r) \end{aligned}$$

Let $X_1 \sim {\text {Geom}}(p_r)$, which is independent of $X_2 \sim {\text {NB}}(r = n, p = p_r)$,

$$\begin{aligned} X&= X_1 + X_2 \\ f_X(k)&= \sum _{i = 0}^{k}f_{X_1}(i)f_{X_2}(k-i) \\&= \sum _{i = 0}^{k} p_r(1 - p_r)^i \left( {\begin{array}{c}k - i + n - 1\\ n - 1\end{array}}\right) p_r^{n}(1 - p_r)^{k - i} \\&= p_r^{n+1}(1 - p_r)^k\sum _{i = 0}^{k}\left( {\begin{array}{c}k - i + n - 1\\ n - 1\end{array}}\right) \\&= p_r^{n+1}(1 - p_r)^k\sum _{i = n}^{k+n}\left( {\begin{array}{c}i - 1\\ n - 1\end{array}}\right) \\&= p_r^{n+1}(1 - p_r)^k \left( {\begin{array}{c}k + n\\ n\end{array}}\right) \\ X&\sim {\text {NB}}(r = n + 1, p = p_r) \end{aligned}$$

where the last identity can be understood combinatorially by considering the selection of *n* unordered seats from a row of k+n total seats. The total number of possible combinations is naturally $$\left( {\begin{array}{c}k+n\\ n\end{array}}\right) $$. Alternatively, we can count the total by partitioning the combinations based on the position of the rightmost chosen seat, denoted by index *i* (where $n \le i \le k+n$). If the rightmost seat is fixed at position *i*, the remaining n-1 seats must be selected from the i-1 available positions to its left, giving $$\left( {\begin{array}{c}i-1\\ n-1\end{array}}\right) $$ possible arrangements for that specific *i*. Because every unique combination has exactly one rightmost seat, this partitioning is strictly mutually exclusive and exhaustive. Summing these arrangements across all valid rightmost positions *i* captures every combination exactly once, proving the equivalence. Thus, for *I* infected individuals, if each of them causes $\Delta E_i \sim {\text {Geom}}(p_r = \frac{\gamma }{\gamma +\beta \frac{S}{N_H}})$

$$\begin{aligned} \Delta E&= \sum _{i=1}^I \Delta E_i \\&= ((\Delta E_1 + \Delta E_2) + \Delta E_3) + \cdots + \Delta E_I \\&= (\underbrace{\Delta E_1 + \Delta E_2}_{{\text {NB}}(r=2)} + \Delta E_3) + \cdots + \Delta E_I \\&= (\underbrace{{\text {NB}}(r=2) + \Delta E_3}_{{\text {NB}}(r=3)}) + \cdots + \Delta E_I \\ \Delta E&\sim {\text {NB}}(r = I, p = p_r) \end{aligned}$$

For each exposed human, the incubation time follows the same exponential waiting-time assumption as the infectious period,

$$\begin{aligned} T&\sim {\text {exp}}(\alpha ). \end{aligned}$$

The probability that an exposed individual becomes symptomatic between $(t_j, t_k)$ is then

$$ p_s^{jk}=e^{-\alpha t_j} - e^{-\alpha t_k}, $$

More specifically, this is the probability that an exposed individual exits the exposed compartment, and hence becomes symptomatic, between times $t_j$ and $t_k$ under the exponential incubation-time assumption. Moreover, parameter $p_h$ represents the probability of hospitalization after becoming symptomatic. Thus, the probability that an exposed individual becomes hospitalized between $(t_i, t_j)$ is

$$ q_i^{jk} = p_hp_s^{jk}, $$

Given the number of newly exposed people $\Delta E_i$ at time $t_i$, the number of hospitalized individuals, $\Delta Y_i^{jk}$, follows a binomial distribution,

$$ \Delta Y_i^{jk} \sim {\text {Binom}}(n = \Delta E_i, p = q_i^{jk}). $$

The binomial form reflects independent thinning of newly exposed individuals according to the probability of becoming symptomatic and hospitalized within the specified time interval. Finally, $\Delta I_i$ newly infected individuals at time $t_i$ will produce $\Delta Y_i^{jk}$ hospitalized individuals between $(t_i, t_j)$, and this compound distribution is also a negative binomial distribution:

$$\begin{aligned} f_{\Delta Y_i^{jk},\Delta E_i}(x)&= \left( {\begin{array}{c}\Delta E_i\\ x\end{array}}\right) (q_i^{jk})^x(1 - q_i^{jk})^{\Delta E_i - x} \left( {\begin{array}{c}\Delta I_i + \Delta E_i - 1\\ \Delta E_i\end{array}}\right) (p_r)^{\Delta I_i}(1 - p_r)^{\Delta E_i} \\ f_{\Delta Y_i^{jk}}(x)&= \sum _{y=0}^{\infty }\frac{(y+x)!}{x!y!} (q_i^{jk})^x(1 - q_i^{jk})^{y} \frac{(\Delta I_i + y + x - 1)!}{(y+x)!(\Delta I_i - 1)!} (p_r)^{\Delta I_i}(1 - p_r)^{y+x} \\&= \frac{(q_i^{jk})^x (p_r)^{\Delta I_i} (1 - p_r)^x}{x!(\Delta I_i - 1)!} \sum _{y=0}^{\infty } \frac{(\Delta I_i + x + y - 1)!}{y!} ((1 - q_i^{jk})(1 - p_r))^{y} \\&= \left( {\begin{array}{c}\Delta I_i + x - 1\\ x\end{array}}\right) (q_i^{jk})^x (p_r)^{\Delta I_i} (1 - p_r)^x \sum _{y=0}^{\infty } \\&\quad \cdot \left( {\begin{array}{c}\Delta I_i + x + y - 1\\ y\end{array}}\right) ((1 - q_i^{jk})(1 - p_r))^{y} \\&= \left( {\begin{array}{c}\Delta I_i + x - 1\\ x\end{array}}\right) (q_i^{jk}(1 - p_r))^x (p_r)^{\Delta I_i} \frac{1}{(1 - (1 - q_i^{jk})(1 - p_r))^{\Delta I_i + x}} \\&= \left( {\begin{array}{c}\Delta I_i + x - 1\\ x\end{array}}\right) (\frac{q_i^{jk}(1 - p_r)}{(1 - (1 - q_i^{jk})(1 - p_r))})^x (\frac{p_r}{(1 - (1 - q_i^{jk})(1 - p_r))})^{\Delta I_i} \\ \Delta Y_i^{jk}&\sim {\text {NB}}\left( r = \Delta I_i, p = \frac{p_r}{p_r + q_i^{jk} - p_rq_i^{jk}}\right) . \end{aligned}$$

However, $\Delta I_i$ is often unknown: the number of newly hospitalized/reported individuals does not reflect the actual number of new infections because newly infected individuals may not all be symptomatic immediately and/or all be hospitalized. To account for these factors, $\Delta I_i$ is approximated by:

$$\begin{aligned} \Delta I_i&= \frac{\Delta Y_i}{p_h(1-e^{-\alpha })}, \end{aligned}$$

where the denominator measures the probability of being symptomatic and hospitalized within a week. In sum, for weekly data, the number of newly reported cases in week *j* can be modeled as

$$\begin{aligned} \Delta Y_j&= \sum _{i = 1}^{j-1} \Delta Y_i^{j(j+1)}, \\ \Delta Y_i^{j(j+1)}&\sim {\text {NB}}(r = \frac{\Delta Y_i}{p_h(1 - e^{-\alpha })}, p = \frac{p_r}{q_i^{j(j+1)}(1-p_r) + p_r}), \\ p_r&= \frac{\gamma }{\gamma + \frac{\beta S}{N_H}}. \end{aligned}$$

#### Base Probabilistic Model with Extrinsic Factors (SEI+T/P/(T+P))

To evaluate the effects of temperature and precipitation on the transmission of CHIKV and Zika, the extended base model allows the transmission rate β to vary with the weekly average temperature *wt* and/or the weekly total precipitation *wp*. Both the CHIKV and the Zika viruses are transmitted via mosquitoes, and the transmission rate is directly related to the blood-feeding frequency and abundance of the adult female mosquitoes. Many field and laboratory studies reported the effects of temperature and precipitation on the gonotrophic cycle (interval between blood-feeding) and mosquito abundance, which suggested the existence of optimal temperature and precipitation levels for different phases of mosquito life cycle. In particular, the immature stages of mosquitoes (e.g. larvae and pupae) have optimal survival at $25-30^\circ$C. Furthermore, the mortality rates will dramatically increase at extreme temperatures (Lee 1994; Waldock et al. 2013). The temperature also impacts the mean duration of the gonotrophic cycle (GC), and the relationship can be described by the following quadratic equation $GC = 56.64 - 3.736\times T + 0.064\times T^2$ (Carrington et al. 2013; Riou et al. 2017), with the optimal value at around $30^\circ$C (Delatte et al. 2009). CHIKV transmissibility at different temperatures in another common mosquito vector called *Aedes albopictus* indicates that CHIKV-carrying mosquitoes have a relatively shorter incubation period and higher transmissibility at $28^\circ$C than at $18^\circ$C, and the adult maturation rate increases significantly between temperatures $25-30^\circ$C (Delatte et al. 2009; Wimalasiri-Yapa et al. 2019). Similarly, low and high precipitation can lead to habitat drying and flushing, which were found to negatively affect the adult breeding activities and survival of larvae, pupae, and adults (Jones et al. 2012; Koenraadt and Harrington 2008; Seidahmed and Eltahir 2016). For these reasons, we assume that the period between disease transmission ($T_{\beta }$) has an optimal value $T_{\beta _o}$ at the optimal temperature and precipitation for mosquito activities such that

$$\begin{aligned} T_{\beta _i}&= T_{\beta _o} + a_t(wt_{i-1} - wt_o)^2 + a_p(wp_{i-1} - wp_o)^2, \\ \beta _i(wt_{i-1}, wp_{i-1})&= \frac{1}{T_{\beta _i}} = \frac{1/T_{\beta _o}}{1 + (a_t/T_{\beta _o})(wt_{i-1} - wt_o)^2 + (a_p/T_{\beta _o})(wp_{i-1} - wp_o)^2}. \end{aligned}$$

Defining $\beta _o = 1/T_{\beta _o}$, ${\text {ct}}_\beta = a_t/T_{\beta _o}$, and ${\text {cp}}_\beta = a_p/T_{\beta _o}$, this simplifies to

3 $$\begin{aligned} \beta _i(wt_{i-1}, wp_{i-1}) = \frac{\beta _o}{1 + {\text {ct}}_\beta (wt_{i-1} - wt_o)^2 + {\text {cp}}_\beta (wp_{i-1} - wp_o)^2}, \end{aligned}$$

where $\beta _o$, $wt_o$, and $wp_o$ represent the optimal transmission rate, optimal temperature, and optimal precipitation. Both temperature and precipitation effects have a one-week delay.

#### Probabilistic Mosquito Model (SEI+M)

Both the CHIKV and the Zika viruses are transmitted via mosquitoes. To explore the effect of mosquito population dynamics on CHIKV and Zika transmission, the base model was extended to include the mosquito population that mediates the spread of disease. The mosquito model consists of two major states: the susceptible mosquitoes ($M_s$) and the infected/infectious mosquitoes ($M_i$). By assumption, $M_s+M_i=N_M$, where $N_M$ represents the total mosquito population size, which remains constant per region. This is a simplifying assumption in the absence of entomological data on mosquito abundance for the study regions.

4 $$\begin{aligned} \frac{dM_s}{dt}&=\; \overbrace{-\beta M_s \frac{I}{N_H}}^{\textit{infection by biting infected humans}} + \overbrace{\phi M_i}^{\textit{turnover (mortality and replacement)}}, \end{aligned}$$

where β denotes the mosquito biting rate and ϕ denotes the per-capita mosquito turnover rate, capturing the combined effect of adult mosquito mortality and replacement by newly emerged susceptible adults; under the constant-$N_M$ assumption, infected mosquitoes that die are replenished by new susceptibles, so 1/ϕ approximates the mean lifespan of an infected mosquito (Yakob and Clements 2013; Poletti et al. 2011; Reinhold et al. 2018). By replacing $M_s$ with $N_M-M_i$, the equation becomes

5 $$\begin{aligned} \frac{dM_i}{dt}&= \beta (N_M - M_i) \frac{I}{N_H} - \phi M_i. \end{aligned}$$

Because mosquito infection turnover (ϕ, on the order of days^-1^) is fast relative to the weekly resolution of the case data, we apply a quasi-steady-state approximation in which $dM_i/dt \approx 0$ within each week. The resulting equilibrium infected-mosquito count is

6 $$\begin{aligned} {M_i}^*=\frac{\beta I_{i-1}{\text {MHr}}}{\phi + \frac{\beta I_{i-1}}{N_H}}, \end{aligned}$$

where ${\text {MHr}}$ is defined as the mosquito-to-human ratio (mosquito density). The same negative-binomial likelihood applies but with $M_i^*$ replacing *I* as the infectious source: the count parameter rescales by $\beta \,{\text {MHr}}/(\phi + \beta I/N_H)$ and the success probability $p_r$ uses mosquito turnover ϕ in place of human recovery γ.

$$\begin{aligned} \Delta Y_j&= \sum _{i = 1}^{j-1} \Delta Y_i^{j(j+1)}, \\ \Delta Y_i^{j(j+1)}&\sim {\text {NB}}\left( r = \frac{\Delta Y_i}{p_h(1 - e^{-\alpha })} \frac{\beta {\text {MHr}}}{\phi + \frac{\beta }{N_H}\frac{\Delta Y_i}{p_h(1 - e^{-\alpha })}}, p = \frac{p_r}{q_i^{j(j+1)}(1-p_r) + p_r}\right) , \\ p_r&= \frac{\phi }{\phi + \frac{\beta S}{N_H}}. \end{aligned}$$

#### Probabilistic Mosquito Model with Extrinsic Factors (SEI+M+T/P/(T+P))

In addition to the gonotrophic cycle and abundance effects discussed in Sect. 2.3.2, temperature also influences the extrinsic incubation period (EIP), the time required for the virus to develop within the mosquito after a viremic blood meal. Johansson et al. model the EIP as $a\times e^{b\times (T-28)}$ (Johansson et al. 2014), where b<0, so temperatures above $28^\circ$C yield shorter EIP (faster viral development) and temperatures below $28^\circ$C yield longer EIP relative to the baseline at $28^\circ$C. By analogy with Eq. (3), we apply the same optimum-response form to both the mosquito biting rate β and the mosquito-to-human ratio ${\text {MHr}}$ in the vector-explicit model, motivated by the same empirical evidence that *Aedes* survival, abundance, and biting activity peak at intermediate temperature and rainfall:

7 $$\begin{aligned} \beta _i(wt_{i-1}, wp_{i-1})&= \frac{\beta _o}{1+{\text {ct}}_\beta (wt_{i-1} - wt_o)^2 + {\text {cp}}_\beta (wp_{i-1} - wp_o)^2} \end{aligned}$$

8 $$\begin{aligned} {\text {MHr}}_i(wt_{i-1}, wp_{i-1})&= \frac{{\text {MHr}}_o}{1+{\text {ct}}_m(wt_{i-1} - wt_o)^2 + {\text {cp}}_m(wp_{i-1} - wp_o)^2} \end{aligned}$$

### Model Comparison, Parameter Estimation and Simulation

Each model is fit to weekly incidence using the negative binomial likelihood specified above. For Bayesian inference, we use *Stan* (HMC/NUTS) to obtain posterior distributions and 95% credible intervals of the parameters (https://mc-stan.org). Convergence is assessed using $\hat{R}$ and effective sample size (ESS) diagnostics, and posterior predictive checks are used to evaluate agreement between model predictions and training data.

Model comparison applies the Akaike Information Criterion (AIC) (Akaike 1973, 1974) to evaluate and compare candidate models. By convention, the AIC is calculated as AIC=-2L+2k, where *L* is the maximized log-likelihood and *k* is the number of parameters. We compute AIC from maximum likelihood estimates using the same negative binomial likelihood. In addition to AIC-based model selection, we further assess predictive performance using the forecasting and validation analyses described later.

**Table 1:** AIC-based model comparison across the eight variants of the modeling framework

Region	Outbreak disease	ΔAIC of each model								Best model
		SEI	SEI+T	SEI+P	SEI+T+P	SEI+M	SEI+M+T	SEI+M+P	SEI+M+T+P	
Austral Islands	Chikungunya	177	194	5150	181	0	5.62	1.63	4.71	SEI+M
	Zika	23.3	28.8	78.8	7.56	9.08	17.6	0	4.59	SEI+M+P
Guadeloupe	Chikungunya	10,000	6690	9490	8110	14,800	23.1	4250	0	SEI+M+T+P
	Zika	6430	2280	4930	4020	6450	3.25	1330	0	SEI+M+T+P
Marquesas Islands	Chikungunya	225	193	201	281	34	55.8	0	10.1	SEI+M+P
	Zika	233	119	117	239	0	8.24	3.15	6.88	SEI+M
Martinique	Chikungunya	33,800	26,800	35,000	21,000	0	42.9	1.38	1.69	SEI+M
	Zika	1310	20,100	2970	1160	10.6	11.8	0	8.58	SEI+M+P
Mo’orea Islands	Chikungunya	226	1270	576	310	1010	8.61	0	14.9	SEI+M+P
	Zika	357	140	288	138	27.2	1.42	5.7	0	SEI+M+T+P
Saint-Martin	Chikungunya	9740	9710	9430	9480	33.3	8.86	0	15.5	SEI+M+P
	Zika	50.3	60.8	51.7	338	0	5.71	15.6	17.6	SEI+M
Sous-le-vent Islands	Chikungunya	935	95.9	34.3	41.3	0	64.1	139	72.2	SEI+M
	Zika	307	45.5	55.7	47.4	69.3	3.86	9.84	0	SEI+M+T+P
Tahiti	Chikungunya	4000	2680	2520	2360	3610	3770	1450	0	SEI+M+T+P
	Zika	15,600	1260	608	2030	44	49.4	0	48.8	SEI+M+P
Tuamotus	Chikungunya	181	191	299	274	8.06	15.7	0	3.07	SEI+M+P
	Zika	49.3	46.9	80.2	61.7	0	5.67	3.07	5.93	SEI+M

Entries report ΔAIC relative to the best model for each outbreak; smaller values indicate better support

## Results

### Model Comparison

To identify the model that best balances parsimony and goodness-of-fit, we calibrated all eight variants of this framework using outbreak data from the nine regions. We then performed model comparison and selection using the AIC test.

Table 1 shows the results of the AIC tests evaluated for outbreak data from different regions. We compute the ΔAIC of each model by subtracting the AIC of the best model from the AIC of the current model. A smaller ΔAIC corresponds to a shorter distance to the best model, and the model with zero ΔAIC is the best. Table 1 shows that incorporating mosquito population dynamics substantially improves predictive accuracy relative to all base models, both with and without extrinsic environmental drivers. This improvement is consistently supported across both diseases and all regions. In contrast, adding temperature effects alone to the base model does not yield a meaningful improvement in predictive performance.

When precipitation effects are incorporated alongside mosquito dynamics, predictive power increases further. This model specification is supported in 12 of 18 outbreaks and is identified as the optimal model in 7 of 18 cases. Adding temperature effects alone to the mosquito-dynamics model improves performance in 6 of 18 outbreaks, although it is never selected as the best model. Notably, incorporating both temperature and precipitation effects in addition to mosquito dynamics yields the strongest overall performance, emerging as the best model in 5 of 18 outbreaks. Taken together, these findings indicate that mosquito dynamics and precipitation are primary determinants of transmission, while temperature contributes additional explanatory power when combined with the other two factors.

**Fig. 3:**
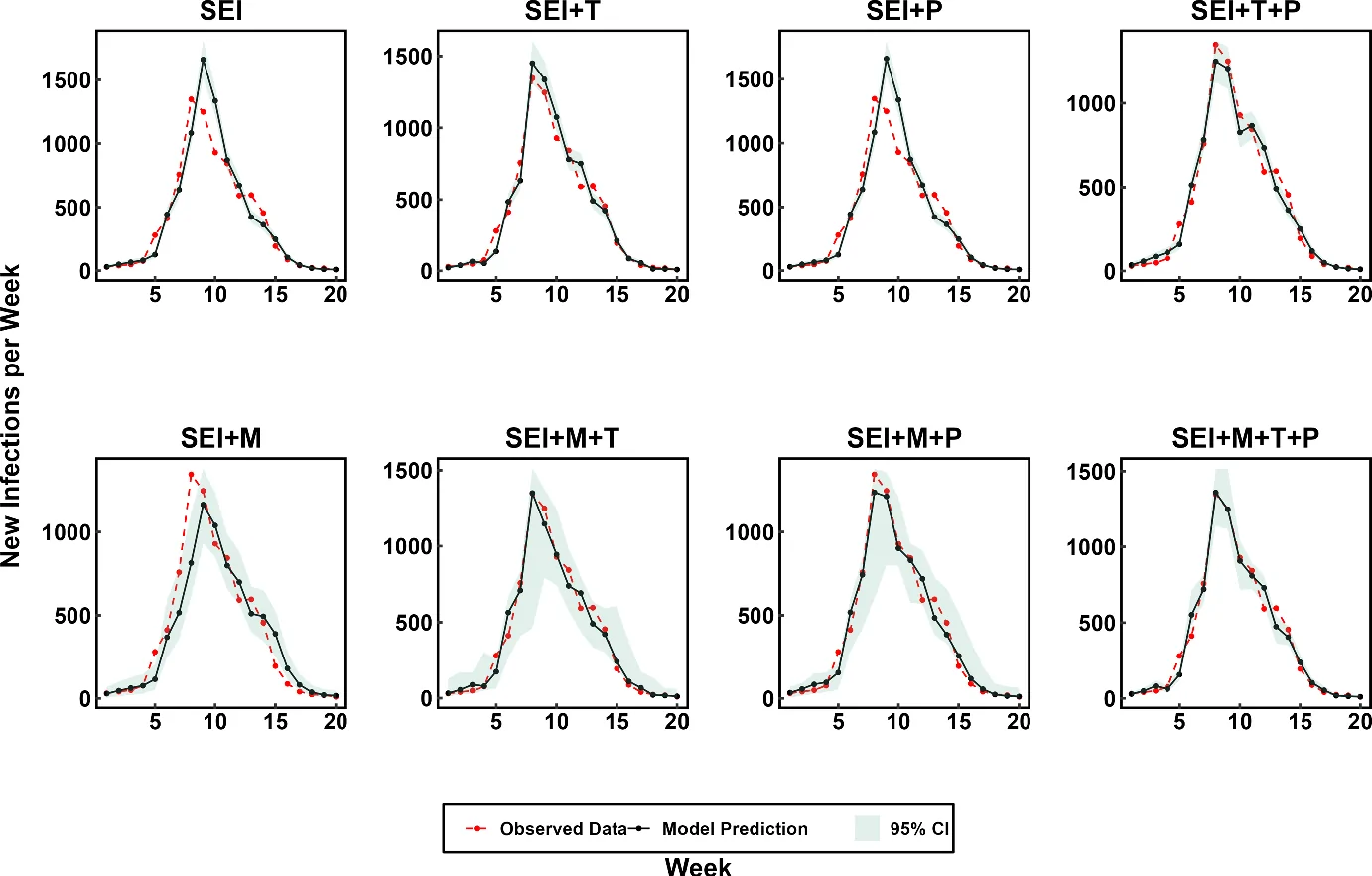
Fits of all eight variants to CHIKV incidence in the Sous-le-vent (SLV) Islands (Color figure online)

Figure 3 illustrates the fitted trajectories of the eight calibrated variants against CHIKV incidence data from the Sous-le-vent Islands. Models that incorporate mosquito dynamics more accurately reproduce both the overall epidemic trajectory and short-term fluctuations. Predictive performance improves further when precipitation and temperature effects are included. Thus, we conclude that mosquito dynamics, precipitation, and temperature are all important factors in the transmission of CHIKV and Zika.

### Model Fitting

**Fig. 4:**
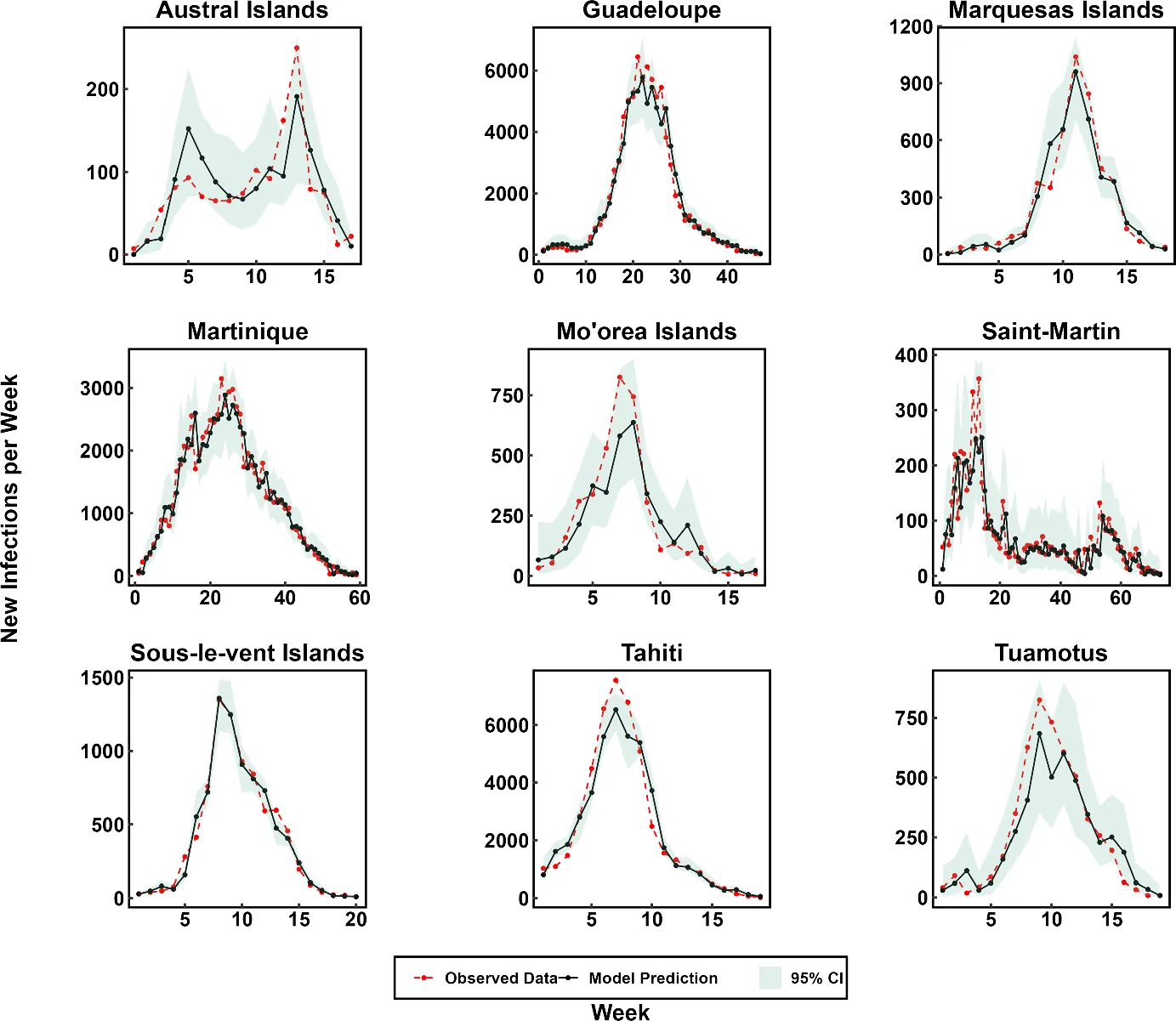
Observed CHIKV incidence and fitted SEI+M+T+P trajectories across regions (Color figure online)

**Fig. 5:**
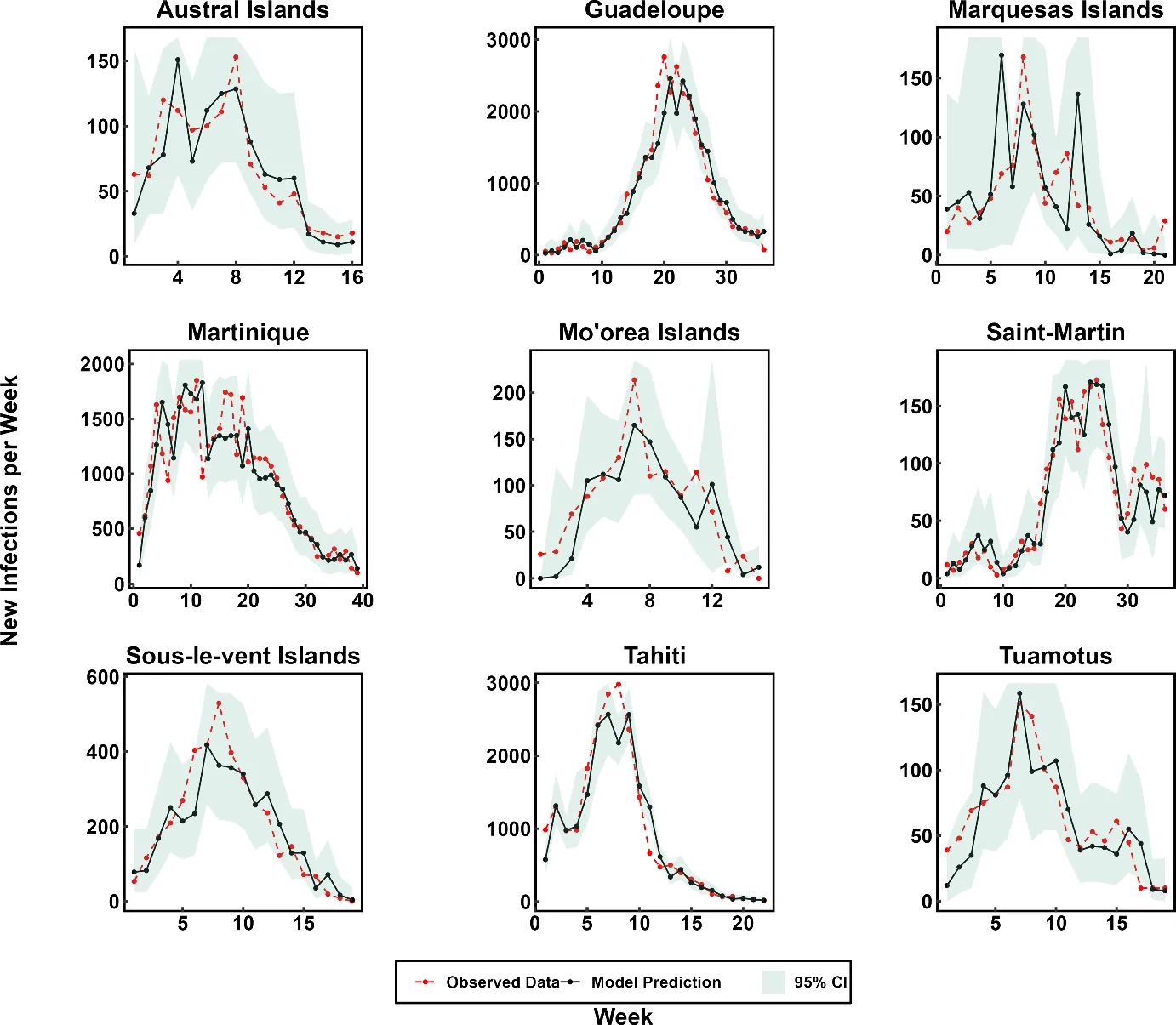
Observed Zika incidence and fitted SEI+M+T+P trajectories across regions (Color figure online)

Consistent with our model comparison results, the Probabilistic mosquito-temperature–precipitation model (SEI+M+T+P) provides significantly better predictions than the alternative models (Figs. 4 and 5). As shown in Figs. 4 and 5, this model accurately predicts the progression, peaks, and termination of both diseases in most regions, indicating that mosquito, temperature, and precipitation are critical factors in disease transmission.

**Fig. 6:**
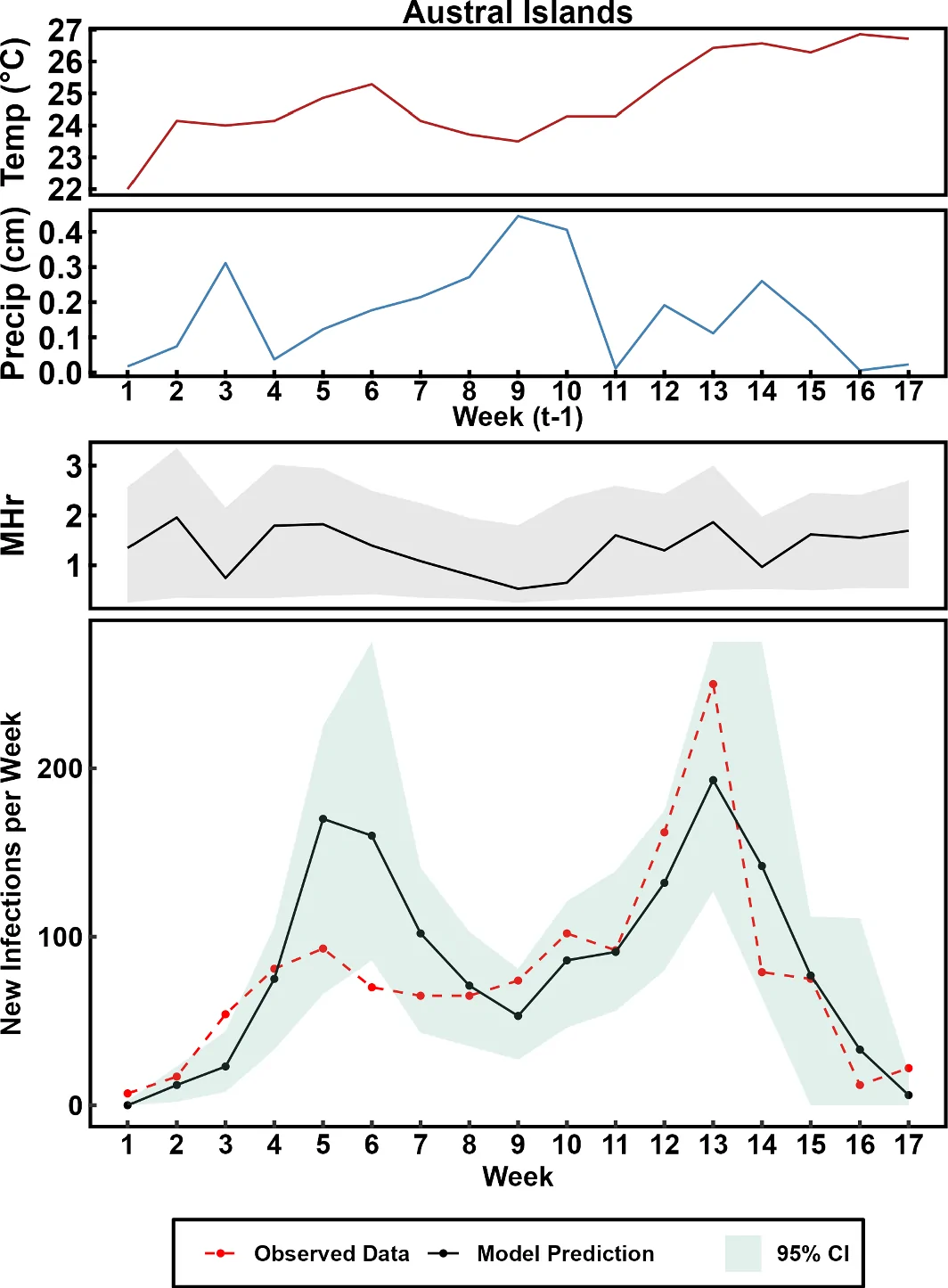
Chikungunya outbreak in Austral Islands: fitted SEI+M+T+P trajectory with weekly average temperature (1-week delay), weekly total precipitation (1-week delay), and model-inferred mosquito-to-human ratio (MHr) dynamics (Color figure online)

In this Austral Islands example (Fig. 6), the major changes in the inferred mosquito-to-human ratio are associated with the lagged precipitation series, but not monotonically; under the fitted model, the remaining shorter-scale fluctuations are induced by temperature variation. The incidence peaks occur during periods of elevated inferred mosquito density.

### Model Validation

#### Forecasting Performance

**Fig. 7:**
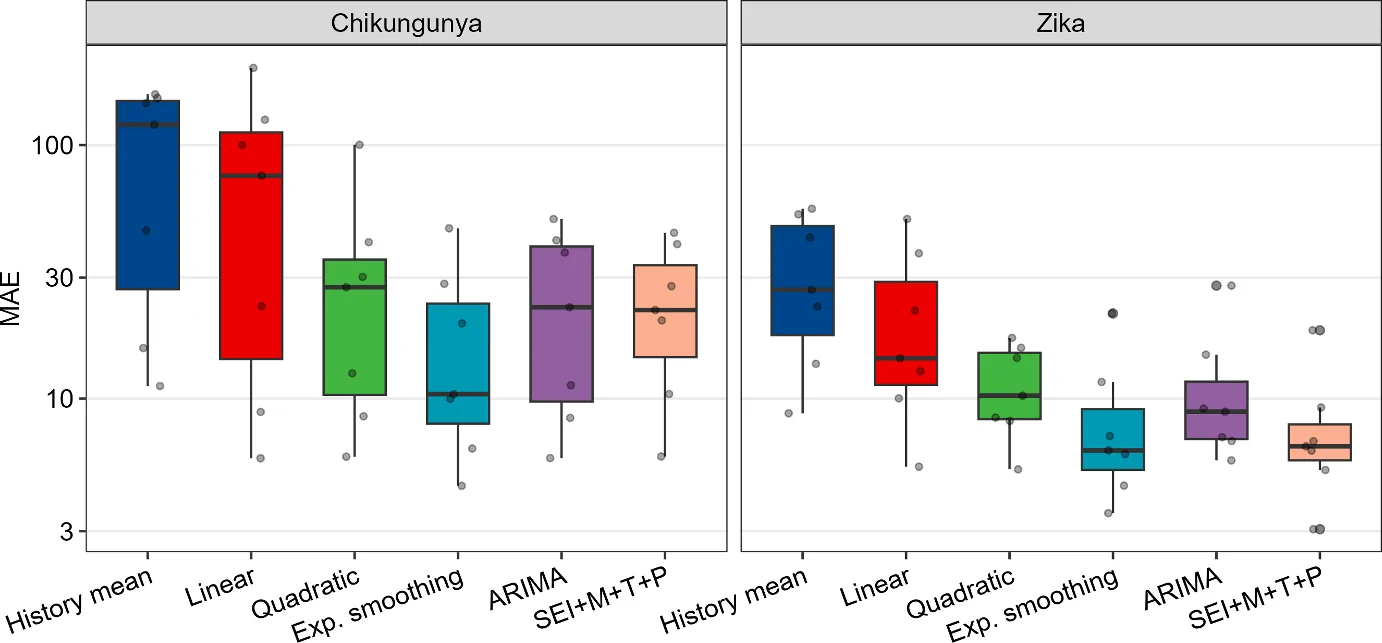
Rolling-window forecast accuracy (MAE) comparing SEI+M+T+P with statistical and baseline forecasters; lower values indicate better performance (Color figure online)

To further validate our mosquito–temperature–precipitation (SEI+M+T+P) model, we evaluated short-term forecasting performance using a rolling-forecast design. Here, ARIMA denotes an autoregressive integrated moving-average benchmark (Box et al. 2015), and exponential smoothing denotes a benchmark that extrapolates future incidence from the recent level and trend of the observed time series (Hyndman et al. 2008). The null baselines use either the historical mean of the training window or simple linear and quadratic autoregressive regressions on recent weekly incidence. Figure 7 summarizes mean absolute error (MAE) across rolling windows under these competing prediction strategies, so lower values indicate smaller out-of-sample prediction errors. Overall, the mechanistic model substantially outperforms the null baselines. Relative to ARIMA/exponential smoothing, it achieves comparable accuracy for Chikungunya and slightly better accuracy for Zika, suggesting that incorporating transmission structure can add predictive value beyond purely autoregressive trend-following.

**Fig. 8:**
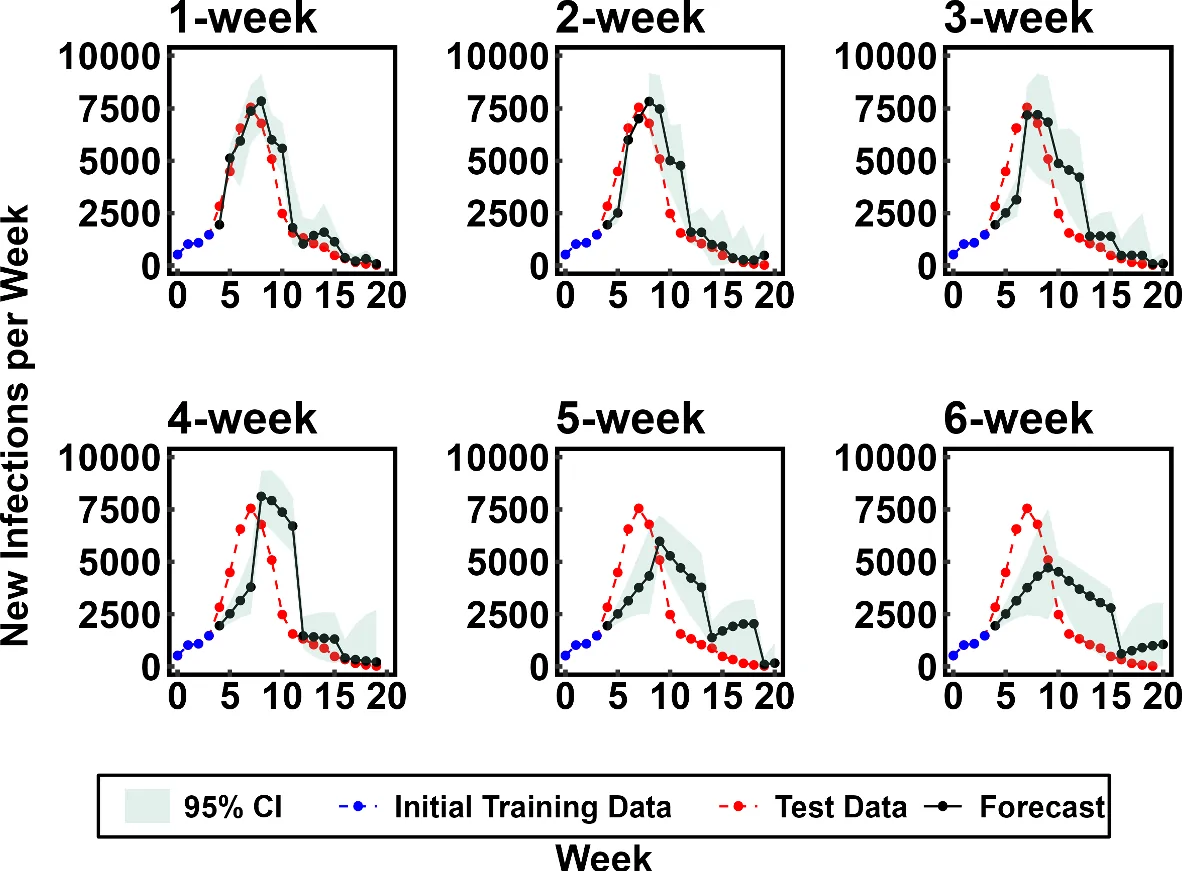
Representative multi-week-ahead forecast from the SEI+M+T+P model against observed incidence (Color figure online)

To complement the aggregate MAE comparison, we next show a multi-week-ahead forecast for a representative outbreak (Fig. 8). Overall, our model can accurately predict the outbreak peak and weekly incidence up to 2–3 weeks ahead.

#### Cross-Disease Validation: Dengue in Brazil

**Fig. 9:**
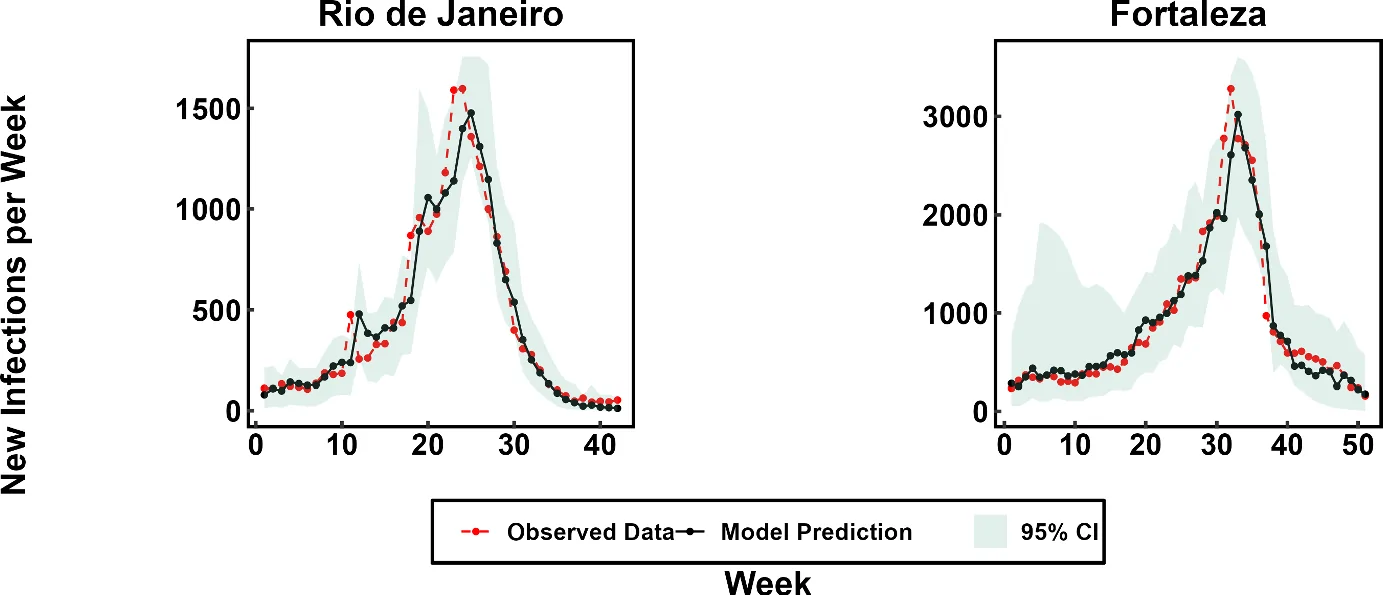
Cross-disease validation: dengue incidence in Brazil with fitted SEI+M+T+P trajectories (Color figure online)

To test model generalizability, we fitted our model (SEI+M+T+P) to the dengue outbreak data from two major cities in Brazil: Rio de Janeiro (2015) and Fortaleza (2016). As shown in Fig. 9, our model accurately predicts the progression of dengue outbreaks in Brazil, suggesting that our framework can potentially be applied to other vector-borne diseases and regions.

### Biological Insights

#### Basic Reproduction Number $R_0$

**Fig. 10:**
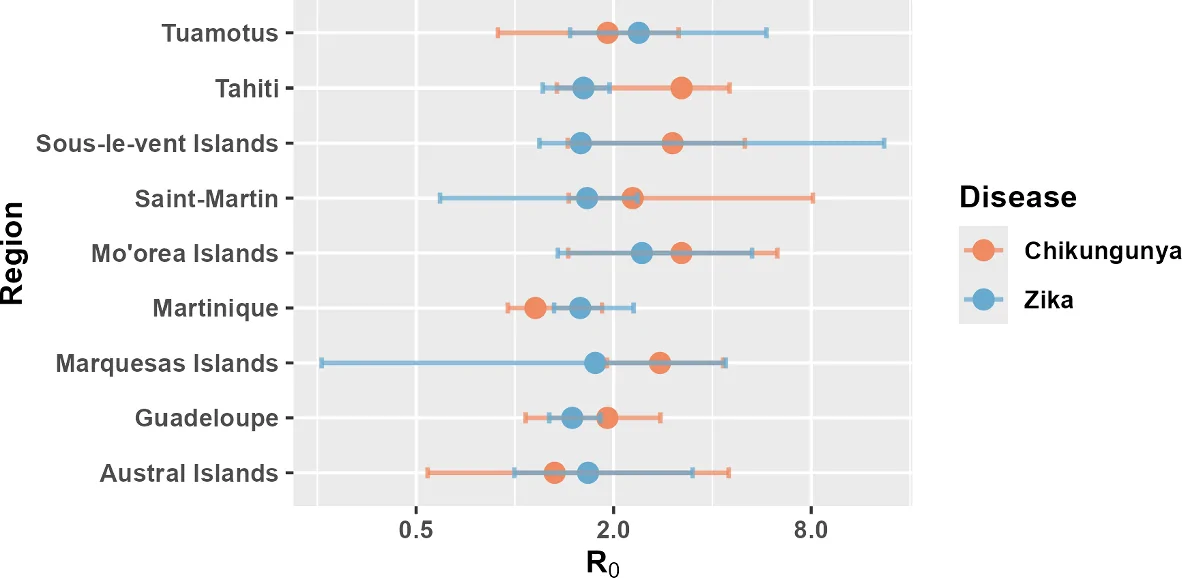
Posterior distributions of $R_0$ by region and disease from the SEI+M+T+P model (Color figure online)

One main goal of this study is to compare the scales of Zika and CHIKV outbreaks among all nine regions and to investigate differences in the transmission dynamics of these two diseases. Our results from the best-fit model (Fig. 10) show that (i) the Zika $R_0$ is similar in each region; (ii) CHIKV often possesses a higher $R_0$ value than Zika; (iii) Marquesas Islands, Mo’orea, Tahiti, and SLV Islands possess significantly higher CHIKV $R_0$ values than other regions. These observations are consistent with the outbreak data shown in Fig. 1. Thus, they serve to support our model: the Zika outbreaks in each region are of a similar scale and smaller than CHIKV outbreaks, and the Marquesas Islands, Mo’orea, Tahiti, and SLV Islands experienced the largest CHIKV outbreaks among all nine regions. Our estimates for CHIKV $R_0$ range from 1.15 to 4.84, with an average of 2.51, and the $R_0$ values for Zika range from 1.49 to 2.44, with an average of 1.80. These values fall within the previously reported ranges of $R_0$ for CHIKV (0.46−6.46) and Zika (0.16−9.4) according to a survey of previous studies (Liu et al. 2020), which shows substantial heterogeneity in $R_0$ across viruses and geographic locations. Riou et al. reported similar average $R_0$ estimates for CHIKV and Zika in these regions (1.82 for CHIKV and 1.87 for Zika) (Riou et al. 2017). The difference between those average estimates and our stronger regional contrast for CHIKV may reflect the additional structure in our model, particularly the explicit mosquito dynamics and the way temperature and precipitation modulate mosquito activity and transmission. Our estimates are also comparable with published estimates for Zika in French Polynesia (3.55 (Hsieh 2017); 1.9 (Nishiura et al. 2016)) and other tropical Pacific regions, such as New Caledonia (1.8 (Champagne et al. 2016)) and Thursday Island, Australia (3.22 (Watson-Brown et al. 2019)).

#### Transmission Parameters

*Aedes aegypti* and *Aedes albopictus* are the two primary vectors responsible for the transmission of both viruses. Although they share some common characteristics, the two mosquito species should contribute differently to the transmission of each disease, as they still differ in the feeding preference, temperature-dependent life cycle, extrinsic incubation period, and transmission ability, inter alia. Furthermore, the prevalence of the two mosquito species could differ significantly among regions, resulting in different outbreak potentials of vector-borne diseases. Due to the lack of species-specific transmission parameter estimates, our model accounts for the overall impact of *Aedes* mosquitoes. Therefore, the model parameters estimated from each fitting only represent a combined effect of both species and thus should be different among regions.

**Fig. 11:**
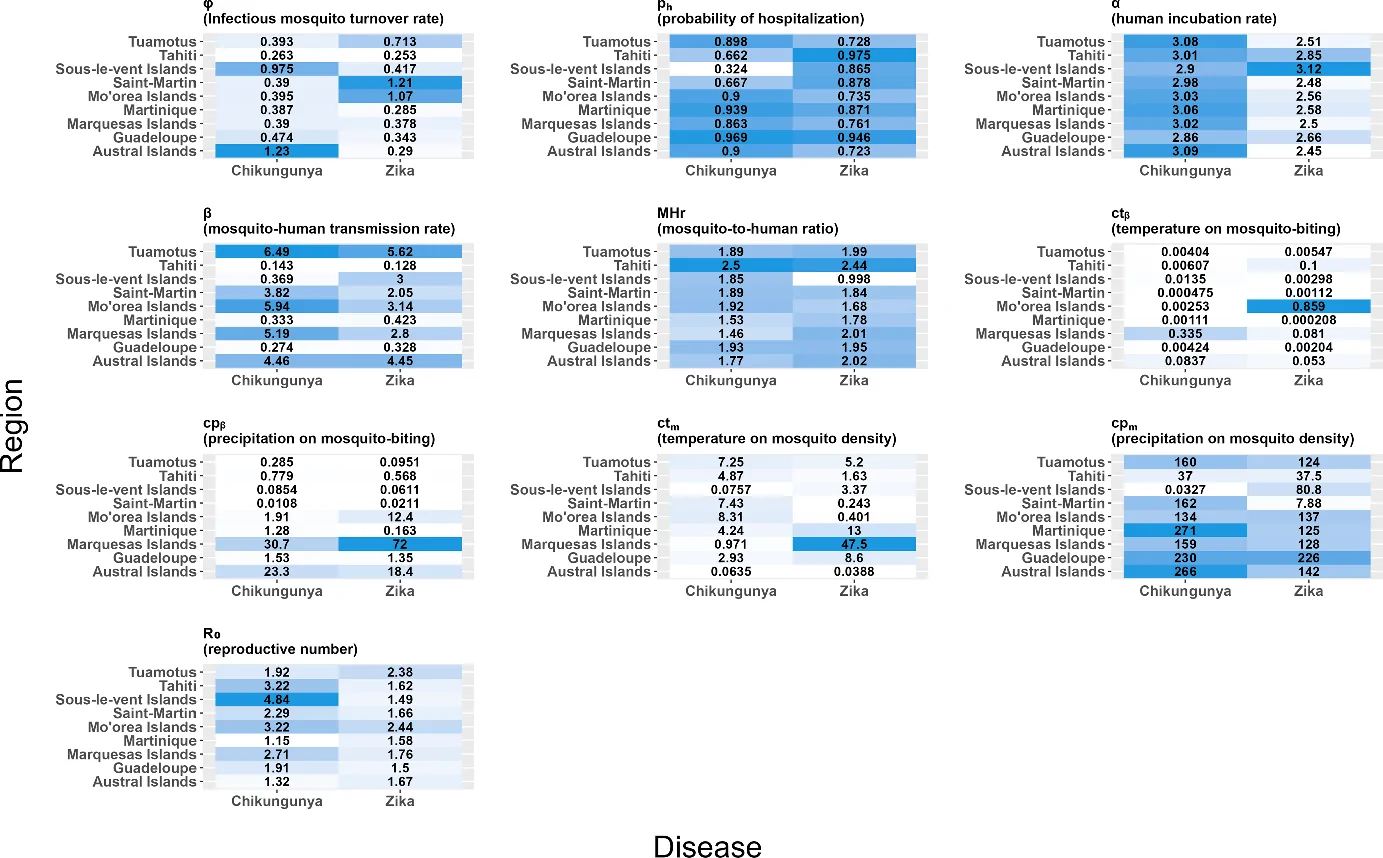
Posterior summaries of key parameters for the SEI+M+T+P model across regions and diseases (Color figure online)

**CHIKV and Zika Outbreak Sizes in the Same Region** Although all nine regions experienced various scales of Zika and CHIKV outbreaks, our parameter estimation (Fig. 11) indicates that the specific reason for the unequal outbreak sizes could differ from region to region. For all the regions and viruses, the mosquito infectious period ($\frac{1}{\phi }$) ranges from 0.5 to 3 weeks, indicating that CHIKV and Zika viruses persist in *Aedes* mosquitoes throughout their lifespans. Although the mosquito infectious periods are similar between CHIKV and Zika, the CHIKV outbreaks were often larger than the Zika outbreaks, suggesting that the larger outbreaks are attributable to other parameters, namely the mosquito-human transmission rate (β) and/or mosquito-to-human ratio (MHr). While the mosquito-to-human ratio, or the mosquito density, is largely region-dependent, the mosquito-human transmission rates β of CHIKV are higher than those of Zika in most regions, which explains the larger outbreak sizes of Chikungunya compared to Zika.

**CHIKV and Zika Outbreak Sizes among Regions** CHIKV outbreaks vary significantly across regions, and among regions with significantly high CHIKV $R_0$s and outbreak sizes, Marquesas, Mo’orea, and Tuamotus have significantly higher mosquito-human transmission rates, suggesting high biting rates and poor mosquito bite prevention in these regions. In contrast, the large CHIKV outbreaks in Tahiti and SLV Islands may be attributable to a combination of moderate transmission rates, temperature, and precipitation conditions that support higher mosquito activity, and lower $p_h$ values, which reduce the fraction of symptomatic individuals removed from transmission through hospitalization. These results suggest that region-specific strategies are needed to control CHIKV outbreaks, such as reducing mosquito abundance in regions with high vector density or reducing human-vector contact in regions with high transmission rates.

The outbreak sizes and $R_0$s of the Zika virus vary much less among all regions. The relatively homogeneous estimates of β suggest that the effective biting rates of mosquitoes transmitting the Zika virus are similar among most regions. Further, the estimates of mosquito density (MHr) for Zika transmission are close in many regions, which indicates the component of mosquitoes that transmit Zika is similar among these areas. These findings suggest that a uniform vector control strategy could be applied universally to all regions to prevent future Zika virus outbreaks.

**Temperature and Precipitation Sensitivities** Our parameter estimation shows that the transmission rate β of CHIKV in Marquesas, Mo’orea, and Austral Islands is highly sensitive to temperature and precipitation changes (${\text {ct}}_\beta$ and ${\text {cp}}_\beta$), which might partially explain the large outbreaks in Marquesas and Mo’orea. Among Zika outbreaks, the disease transmission rate in Mo’orea has the highest sensitivity to temperature fluctuations (${\text {ct}}_\beta$).

In terms of mosquito density, the mosquito populations for transmitting CHIKV are more sensitive to precipitation than those for transmitting Zika virus (${\text {cp}}_{\text {m}}$). In contrast, the temperature sensitivity of mosquito density depends on both virus and region (${\text {ct}}_{\text {m}}$).

These findings indicate that CHIKV and Zika transmission strongly depend on temperature and precipitation, and their vector populations depend differentially on these two factors.

**Fig. 12:**
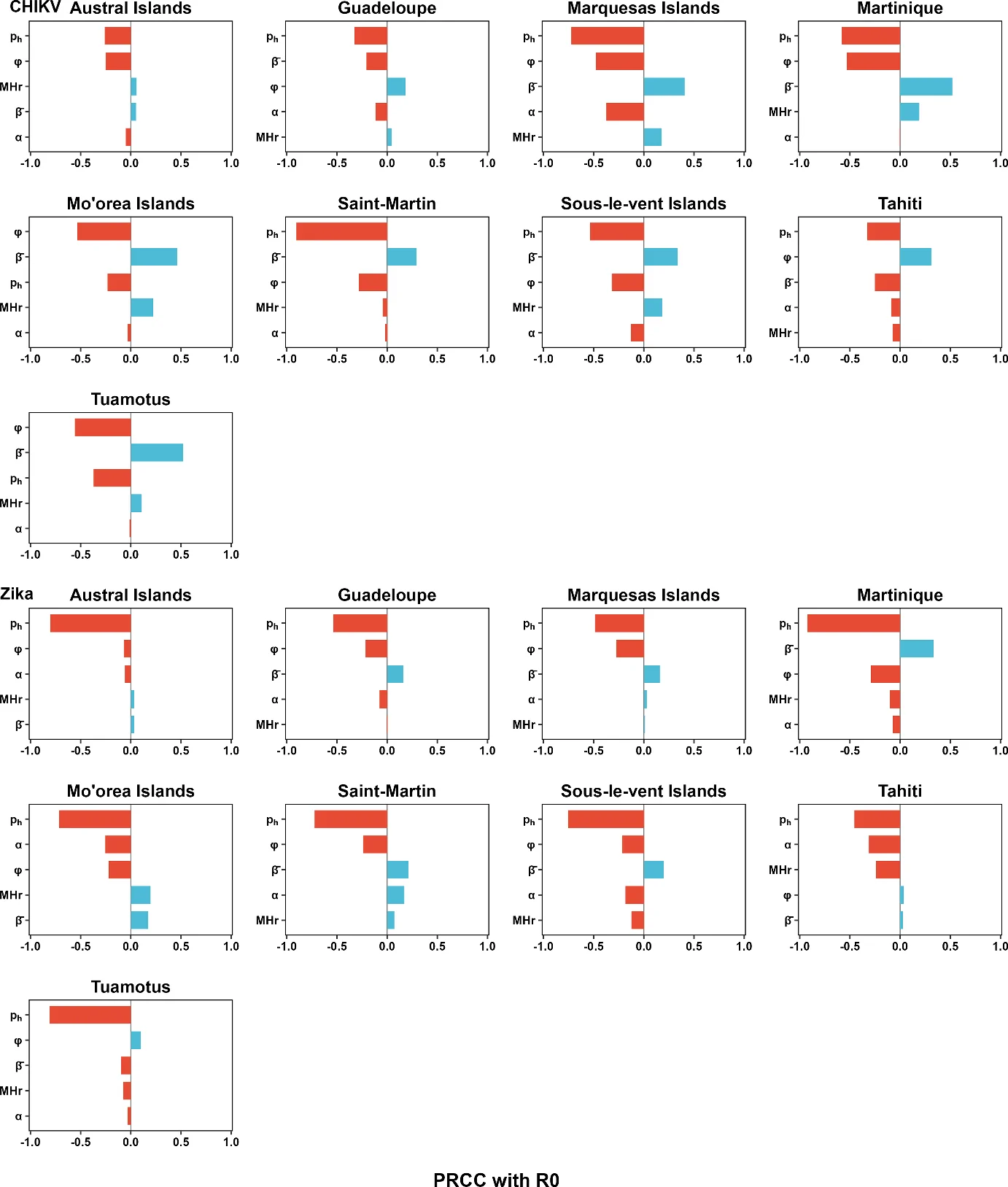
Global sensitivity analysis (PRCC) of $R_0$ with respect to key parameters for CHIKV and Zika across regions (Color figure online)

**Sensitivity analysis** To identify the most important factors that affect transmissibility of each disease in each region, we conducted global sensitivity analysis by computing the partial rank correlation coefficient (PRCC) between key model parameters (α, β, $p_h$, ϕ, MHr) and $R_0$ (Fig. 12). In most regions, the probability of hospitalization is the most sensitive parameter for reducing $R_0$, suggesting that human behavior, especially self-isolation after becoming infected, is likely the most effective way to reduce transmission. On the other hand, mosquito activity and turnover seem to be equally important drivers of Chikungunya transmission in the Marquesas Islands, Martinique, Mo’orea, and the Tuamotus, suggesting the importance of vector control in these areas.

## Discussion

This study evaluated the drivers of CHIKV and Zika transmission in French Polynesia and the French West Indies by comparing a group of mechanistic models that incorporate vector dynamics and environmental forcing. Our comparison suggests that a parsimonious model integrating mosquito dynamics with precipitation and temperature (SEI+M+T+P) balances mechanistic realism with predictive power. This model is supported by favorable AIC performance across most regions, by forecasting accuracy comparable to standard statistical benchmarks like ARIMA, and by its ability to fit out-of-sample data, such as dengue outbreaks in Brazil. These results indicate that the proposed framework can potentially be applied to investigate arboviral transmission beyond the specific context of French Polynesia.

By dissecting the roles of intrinsic versus extrinsic drivers, our analysis highlights that incorporating mosquito dynamics improves model fit; purely human-to-human transmission models failed to capture the characteristic shape of these outbreaks. Among environmental factors, precipitation appears to be a primary driver of transmission variability, improving model fit in the majority of cases. In the tropical setting of French Polynesia, where temperatures remain consistently warm ($25-30^\circ$C) and near the thermal optimum for *Aedes* vectors (Mordecai et al. 2017), temperature likely acts as a constant enabler of transmission rather than a limiting factor. Precipitation variability, by contrast, modulates the availability of aquatic breeding sites, serving as a variable switch that regulates vector abundance and, consequently, outbreak magnitude. The full SEI+M+T+P model captures this synergy, with precipitation driving the potential for population growth and temperature modulating transmission rates.

Beyond model fitting, these results illustrate the potential of mechanistic modeling for forecasting. While statistical models are effective at projecting trends based on autocorrelation, they often function as “black boxes” that cannot anticipate turning points driven by external shocks. Our mechanistic approach provides “white-box” forecasts that explicitly link future incidence to observed environmental conditions, such as heavy rainfall events. Since our model matches the predictive accuracy of ARIMA (Fig. 7) and provides 2–3 weeks of accurate forecasts, it can potentially be a useful forecasting tool for public health. The cross-disease fitting on dengue data from Brazil further suggests that this model structure may be adaptable to other *Aedes*-borne pathogens and geographic regions.

The framework may also be useful in regions with larger seasonal variation, such as the southern United States or parts of Europe, where transmission is more strongly constrained by colder temperatures and broader climate variability. In such settings, the same compartmental structure could be retained, but the model would likely require re-parameterization and potentially additional environmental covariates, such as humidity effects.

Our parameter inference reveals distinct transmission ecologies for Zika and CHIKV. Zika outbreaks were characterized by relatively homogeneous $R_0$ values and transmission parameters across regions, suggesting a uniform “baseline” transmissibility that might be managed with standardized vector control strategies. In contrast, CHIKV outbreaks exhibited significant heterogeneity. For example, the large outbreaks in the Marquesas Islands were associated with high mosquito-to-human transmission rates (β), possibly reflecting efficient biting behavior or local vector competence, whereas outbreaks in other regions appeared to be driven more by high vector density (MHr). This heterogeneity implies that controlling CHIKV may require region-specific strategies, where some islands benefit most from reducing mosquito abundance (larvicides) while others require interventions to reduce human-vector contact (bed nets, repellents).

From a control perspective, our global sensitivity analysis indicates that the probability of hospitalization ($p_h$) is a critical parameter for reducing $R_0$ across most regions and viruses. Since hospitalization in our model serves as a proxy for the isolation of symptomatic individuals, this finding suggests that reducing human-to-mosquito transmission through rapid diagnosis and isolation could be an effective complement to vector control. Therefore, a dual strategy that combines vector control with timely case isolation may offer robust protection against future arbovirus outbreaks.

Limitations of this study include the lack of longitudinal entomological data to directly validate the inferred mosquito population dynamics. While our “M” compartment improves fit, it remains a latent variable inferred from human incidence. Additionally, our model assumes spatial homogeneity within regions, ignoring the potential role of human mobility or local clusters in sustaining transmission. Although human population size $N_H$ is included in the transmission terms, population density is not modeled separately; density-related effects on mosquito-human contact are therefore absorbed into effective parameters such as β and MHr. Another limitation is that we did not explicitly consider viral strains across outbreaks. Different CHIKV or Zika strains may differ in transmissibility, human incubation period, human infectious period, or mosquito infectious period, which could affect parameters such as β, α, γ, and ϕ. Because strain-level data were not included in this analysis, these effects are not separated from regional differences in the model. Future work should aim to integrate real-time vector surveillance and human mobility data to further refine these mechanistic forecasts.

## Figures and Tables

**Fig. 13:**
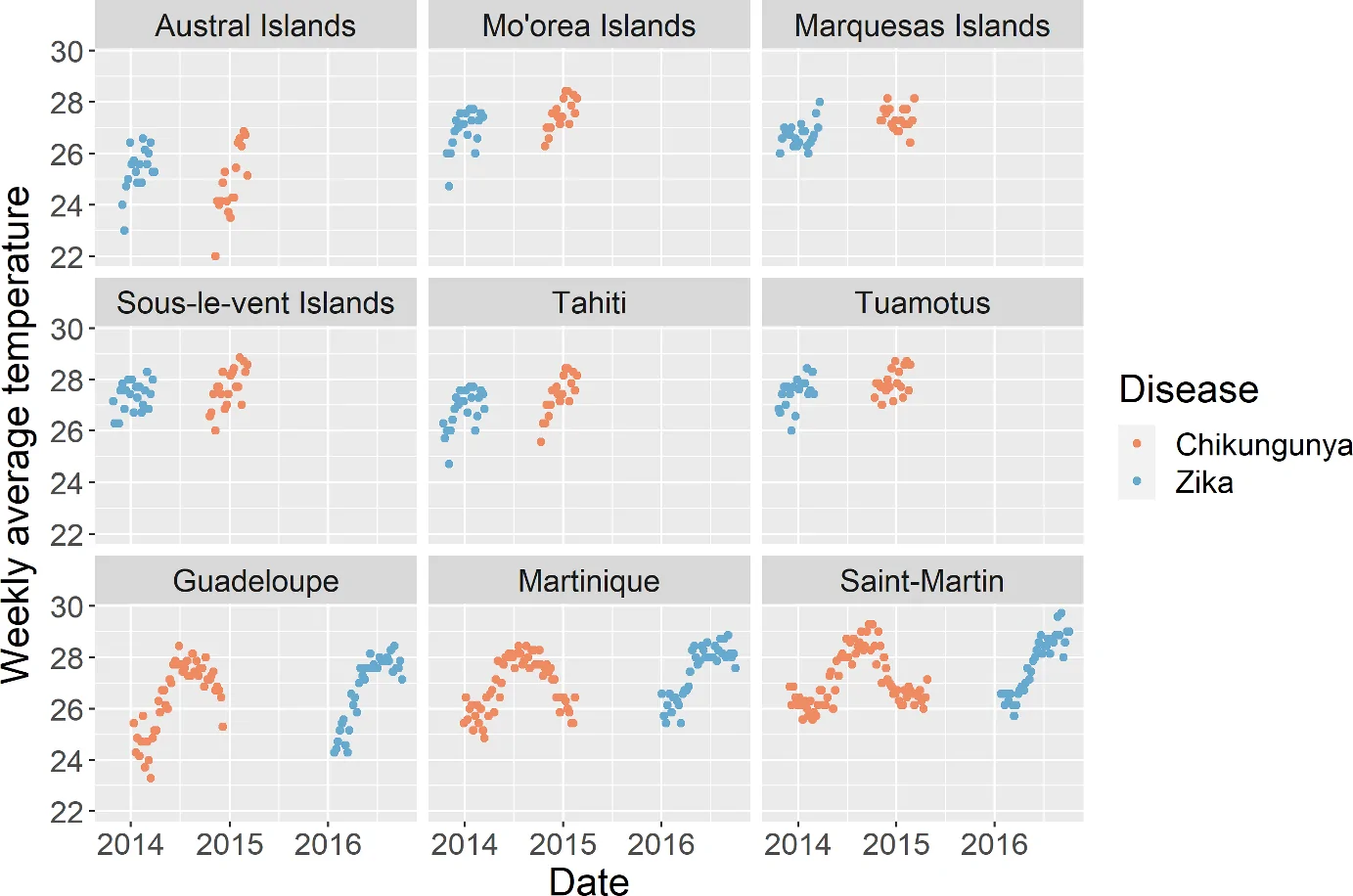
Weekly mean temperature during outbreak periods across the nine study regions (six in French Polynesia and three in the French West Indies). Points show weekly values for CHIKV *(orange) and Zika (blue)* (Color figure online)

**Fig. 14:**
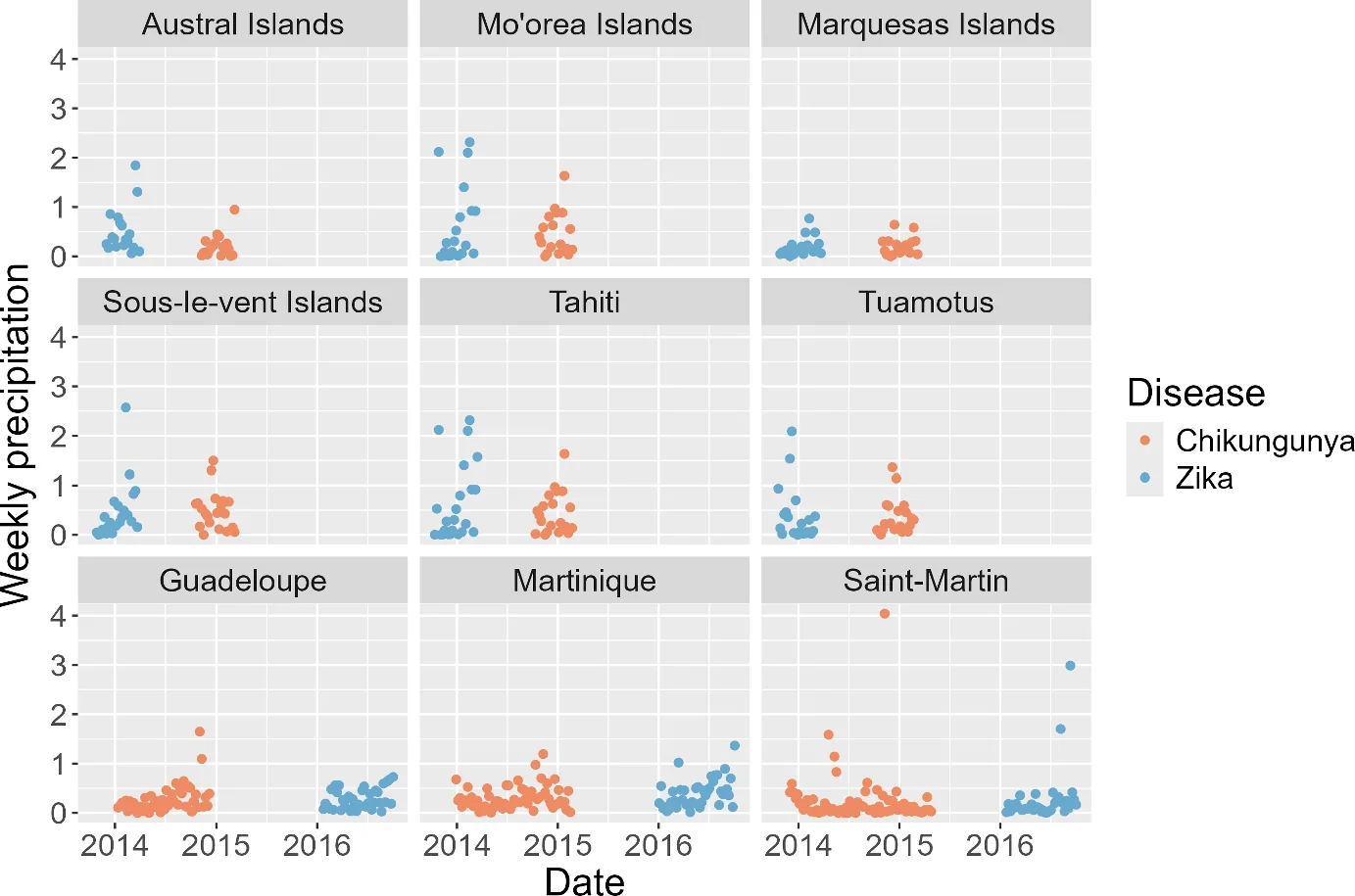
Weekly total precipitation during outbreak periods across the nine study regions (six in French Polynesia and three in the French West Indies). Points show weekly values for CHIKV *(orange) and Zika (blue)* (Color figure online)

## Data Availability

The incidence and weather data used in this study are publicly available from Riou et al. (2017). The dengue data from Brazil were retrieved from InfoDengue (InfoDengue Project 2026). Code for reproducing the analyses is available at https://github.com/stanfish06/Chikungunya-Zika-paper-code-2026.

## Appendix A

See Figs. 13 and 14.

## Appendix B

To derive the probability distribution for the number of new infections ($E_{\text {new}}$) caused by a specific group of infectious individuals ($I_0$), we set up the continuous-time Markov chain (CTMC) with discrete states (i.e. to account for the discrete nature of the incidence data) using the same relevant transitions as in the continuous-time SEIRH ODE model. We treat *S*/*N* as approximately constant during each inter-event interval, since the number of new exposures per infectious period is small relative to *N*:

$$\begin{aligned} \emptyset&\xrightarrow {\beta I \frac{S}{N}} E, \\ I&\xrightarrow {\gamma I} \emptyset , \\ E(0)&= 0, \\ I(0)&= I_0. \end{aligned}$$

To track the evolution of probability distributions of *E* and *I* over time, we convert the transitions defined above to the corresponding master equations:

$$\begin{aligned} \frac{dp^{E}_{i}}{dt}&= \beta I \frac{S}{N}(p^{E}_{i-1} - p^{E}_{i}), \\ \frac{dp^{I}_{i}}{dt}&= \gamma (i+1) p^{I}_{i+1} - \gamma i p^{I}_{i}, \\ p^{E}_0(t = 0)&= 1, \\ p^{I}_{I_0}(t = 0)&= 1, \\ E \in \mathcal {X}_E&= \{0,1,2,...,N\}, \\ I \in \mathcal {X}_I&= \{0,1,2,...,I_0\}. \\ \end{aligned}$$

To solve the master equations and obtain $p^{E}_i(t)$ and $p^{I}_i(t)$, we first solve the inter-event time $T_I$ for *I*, whose cumulative distribution function and density follow

$$\begin{aligned} G(t)&= {\text {Prob}}(T_I > t), \\ G(t + \Delta t)&= {\text {Prob}}(I(t + \Delta t) = i | I(t) = i) G(t), \\&= (1 - \gamma i \Delta t + o(\Delta t)) G(t), \\ \frac{dG}{dt}&= - \gamma i \, G(t), \\ F(t)&= 1 - G(t) = 1 - e^{(- \gamma i t)}, \\ T_I&\sim {\text {Expo}}(\gamma i). \end{aligned}$$

Next, we solve $p^{E|I=j}_{i}(t)$, the conditional probability distribution of *E* given I=j. The probability generating function for E|I=j is

$$\begin{aligned} P_{E|I=j}(z, t)&= \sum _{k=0}^\infty {p^{E|I=j}_{k}(t)z^k}, \\ \frac{dP_{E|I=j}(z, t)}{dt}&= \sum _{k=0}^\infty {\frac{p^{E|I=j}_{k}(t)}{dt}z^k}, \\&= \sum _{k=1}^\infty {\beta j \frac{S}{N}(p^{E|I=j}_{k-1} - p^{E|I=j}_{k})z^k} - \beta j \frac{S}{N}p^{E|I=j}_{0}, \\&= z\beta j\frac{S}{N}\sum _{k=0}^\infty {p^{E|I=j}_{k}z^k} - \beta j\frac{S}{N}\sum _{k=0}^\infty {p^{E|I=j}_{k}z^k}, \\&= -\beta j\frac{S}{N}P_{E|I=j}(z, t)(1 - z), \\ P_{E|I=j}(z, t)&= e^{(-\beta jt\frac{S}{N}(1 - z))}. \end{aligned}$$

Therefore, $E|I=j \sim {\text {Poiss}}(\beta jt\frac{S}{N})$, which holds for $t \le T_{I=j}$ amount of time. After marginalizing over $T_{I=j}$, the final distribution is geometric:

$$\begin{aligned} E|I=j(T_{I=j})&\sim {\text {Geom}}\left( p = \frac{\gamma j}{\gamma j + \beta j\frac{S}{N}}\right) , \\&\sim {\text {Geom}}\left( p = \frac{\gamma }{\gamma + \beta \frac{S}{N}}\right) , \end{aligned}$$

which no longer depends on I=j nor $T_{I=j}$. Because *j* cancels, each infectious individual independently contributes a geometric number of new exposures with the same success probability. The sum of $I_0$ i.i.d. geometric random variables gives

$$\begin{aligned} E\left( \sum _{k=1}^{I_0}T_{I=k}\right)&= \sum _{k=1}^{I_0}{E|I=k(T_{I=k})}, \\&\sim {\text {NB}}\left( r=I_0, p=\frac{\gamma }{\gamma + \beta \frac{S}{N}}\right) . \end{aligned}$$

Thus, $I_0$ infectious individuals eventually give a negative binomial number of newly exposed individuals.
